# Demographic history and conservation genomics of caribou (*Rangifer tarandus*) in Québec

**DOI:** 10.1111/eva.13495

**Published:** 2022-10-25

**Authors:** Morgan N. Dedato, Claude Robert, Joëlle Taillon, Aaron B. A. Shafer, Steeve D. Côté

**Affiliations:** ^1^ Environmental and Life Sciences Graduate Program Trent University Peterborough Ontario Canada; ^2^ Département des Sciences Animales Université Laval Québec Québec Canada; ^3^ Direction de l'expertise sur la Faune Terrestre, l'herpétofaune et l'avifaune, Ministère des Forêts, de la faune et des parcs Gouvernement du Québec Québec Québec Canada; ^4^ Forensics Department Trent University Peterborough Ontario Canada; ^5^ Département de Biologie, Caribou Ungava and Centre d'Études Nordiques Université Laval Québec Québec Canada

**Keywords:** ancestral varaiation, effective population size, genetic diversity, threatened, ungulates

## Abstract

The loss of genetic diversity is a challenge many species are facing, with genomics being a potential tool to inform and prioritize decision‐making. Most caribou (*Rangifer tarandus*) populations have experienced significant recent declines throughout Québec, Canada, and are considered of concern, threatened or endangered. Here, we calculated the ancestral and contemporary patterns of genomic diversity of five representative caribou populations and applied a comparative population genomics framework to assess the interplay between demographic events and genomic diversity. We first calculated a caribou specific mutation rate, *μ*, by extracting orthologous genes from related ungulates and estimating the rate of synonymous mutations. Whole genome re‐sequencing was then completed on 67 caribou: from these data we calculated nucleotide diversity, *θ*_*π*_ and estimated the coalescent or ancestral effective population size (*N*_e_), which ranged from 12,030 to 15,513. When compared to the census size, *N*_C_, the endangered Gaspésie Mountain caribou population had the highest ancestral *N*_e_:*N*_C_ ratio which is consistent with recent work suggesting high ancestral *N*_e_:*N*_C_ is of conservation concern. In contrast, values of contemporary *N*_e_, estimated from linkage‐disequilibrium, ranged from 11 to 162, with Gaspésie having among the highest contemporary *N*_e_:*N*_C_ ratio. Importantly, classic conservation genetics theory would predict this population to be of less concern based on this ratio. Interestingly, *F* varied only slightly between populations, and despite evidence of bottlenecks across the province, runs of homozygosity were not abundant in the genome. Tajima's *D* estimates mirrored the demographic models and current conservation status. Our study highlights how genomic patterns are nuanced and potentially misleading if viewed only through a contemporary lens; we argue a holistic conservation genomics view should integrate ancestral *N*_e_ and Tajima's *D* into management decisions.

## 1 INTRODUCTION

Human disturbances, including habitat alterations and climate change, are causing many wildlife populations to decline, generating concern over the maintenance of biodiversity and ecosystem health (Ceballos et al., 2010; Johnson et al., 2015). Species requiring a lot of space to maintain vital activities such as foraging and predator avoidance are vulnerable to human development (Plante et al., 2020). Of the five assessment criteria used by the International Union for Conservation of Nature (IUCN), population size is the first listed (IUCN Standards and Petitions Committee, 2019). Across taxa, however, there is a disconnect between genetic diversity and population size – referred to as the Lewontin's paradox – such that diversity metrics like nucleotide diversity (π) vary substantially less compared to census size or *N*_C_ (Ellegren & Galtier, 2016; Lewontin, 1974). Demographic history, genetic drift, mutation rate and linked (background) selection all influence diversity (Charlesworth, 2009; Lynch et al., 2016), with each of these effects occurring over a varying number of generations, creating a time‐lag between genetic diversity estimates and fluctuations of *N*_C_. Quantifying these factors is key to understanding the relationship between diversity and population size (Ellegren & Galtier, 2016) and aids in targeted mitigation plans for management and conservation.

Large migratory mammals are considered irreplaceable components of Canada's biodiversity; however, many migratory species are affected by rapid anthropogenic development across the boreal and subboreal forests causing population declines across Canada (Johnson et al., 2015). This loss of individuals means populations are at a greater risk of losing genetic diversity via genetic drift (Wright, 1931). Reduced genetic variation is typically representative of declining populations and is thought to limit the capacity of individuals to adapt and evolve relative to different and changing environmental conditions (Frankham, 1996; Lande, 1995). Specifically, each population's intraspecific genetic variation plays a role in the population persistence during numerical (i.e. census size) declines (Stoffel et al., 2018). In contrast to *N*_C_, *N*_e_ represents the number of breeding individuals in an idealized population that would lose genetic variation at the same rate as the observed population or *N*_C_ (Wright, 1931). Broadly speaking, *N*_e_ is an estimate of genetic diversity under Kimura's neutral model of evolution nucleotide diversity (*θ*_π_) scales according to *N*_e_ (Gillespie, 2004). Contemporary *N*_e_ can be calculated using a variety of non‐genetic and genetic methods (Frankham, 1995), with non‐genetic approaches often depending on demographic and pedigree data and produce short‐term estimates over a single or few generations (Frankham, 1995; Leroy et al., 2013). Genetic estimates of *N*_e_ can be inferred from heterozygosity excess and temporal allele sampling (Wang, 2005). Measures of linkage disequilibrium between independently segregating sites also scale inversely proportional to contemporary *N*_e_ (Waples et al., 2016), and are among the most common algorithms used (Do et al., 2014).

The same genetic data also provide the information required for the ancestral or coalescent *N*_e_ (Wakeley & Bell, 2010), which can be derived simply by solving the equation: *θ*_*π*_ *= 4N*_*e*_ *μ* (Kimura, 1969). While nucleotide diversity or *θ*_π_ can be readily estimated by comparing at least two chromosomes, this approach requires knowing the lineage‐specific mutation rate, *μ*, though Peart et al. (2020) argued using closely related species values was appropriate. The coalescent *N*_e_ scales with the harmonic mean of *N*_e_ of each generation, back to the most recent common ancestor (often millions of years). Therefore, it encapsulates a variety of factors that have shaped the ancestral genetic variation that is still observed in the current population census (Wakeley & Bell, 2010).

The relationship between contemporary *N*_e_ and *N*_C_ can be a predictor of the population adaptive potential (Frankham, 1995). Under neutrality, *N*_e_ should scale proportionally with *N*_C_, making the ratio a critical parameter for wildlife management as you can predict one from the other (Frankham, 1995). The potential of the population to sustain disturbances, and recover in the future can be informed by quantifying *N*_e_/*N*_C_ relationship and factors affecting it (Franklin, 1980). For example, a low contemporary *N*_e_/*N*_C_ ratio reflects decreased diversity within the population and limits the capacity for individuals to respond to selection (Palstra & Fraser, 2012), though there is no clear link to conservation status (Palstra & Ruzzante, 2008). In contrast, contemporary population declines due to recent anthropogenic activity are not necessarily expected to leave a visible impact in the estimates of ancestral *N*_e_ (Peart et al., 2020), meaning higher genomic diversity might be retained from the ancestral population than inferred from the contemporary *N*_e_ approaches. Indeed, high ancestral *N*_e_:*N*_C_ ratios correlated to IUCN threat status in marine mammals (Peart et al., 2020). Populations therefore might be capable of rebounding after a sharp decline (such as a bottleneck) if individuals harbor enough genome‐wide diversity through the decline (Stoffel et al., 2018). Of note, Tajima's *D* – a statistic derived from *θ*_π_ and Wattersons *θ* – correlates well to population trajectories inferred from explicit demographic models and the ancestral *N*_e_ *:N*_C_ relationship (Peart et al., 2020), making it useful for understanding and predicting how wild populations respond to selective pressures.

Past population bottlenecks can have negative genetic consequences due to drift and inbreeding (Lande, 1994; Mills & Smouse, 1994), but the key parameter dictating genetic diversity is the length of time in the bottleneck (Gillespie, 1998). Thus elevated inbreeding coefficient, *F*, and runs of homozygosity (ROH) might not accrue if the bottleneck is short or very recent: in fact changes in inbreeding depression and genetic load may often be negligible and return back to equilibrium, dependent on the size of the bottleneck and speed of population growth (Kirkpatrick & Jarne, 2000). In Eastern Canada, most caribou (*Rangifer tarandus*) populations that were once historically distributed throughout Québec and neighbouring provinces are declining in *N*_C_ (Festa‐Bianchet et al., 2011). This iconic species is relied upon northern and Indigenous communities for food and cultural practices (Festa‐Bianchet et al., 2011). Three ecotypes of caribou are present in Eastern Canada: (1) migratory caribou that move throughout northern Labrador and Québec, (2) sedentary or boreal caribou that reside in the boreal forest and (3) mountain caribou, including one population south of the St. Lawrence River in the Gaspé Peninsula and the other one in the Torngat Mountains (Yannic et al., 2016). Declines in these populations that recently occurred or are undergoing, are mainly due to direct and indirect human activities (Festa‐Bianchet et al., 2011) and could put them at genetic risk (Frankham, 1996). Here, we implemented a comparative population genomics approach to quantify the relationship between genomics parameters, demographic history, and conservation status of caribou. Using genomic data from five populations, we explored the interplay between demographic trends (i.e. explicit models and Tajima's *D*), genetic diversity (*F* and ROH) and the *N*_e_/*N*_C_ ratio (ancestral and contemporary), and compared the results among ecotypes to characterize population history and their current trajectory.

## 2 METHODS

### 2.1 Obtaining orthologs and estimating *μ*

We estimated a caribou specific mutation rate using branch‐specific substitution rates of synonymous coding sequence sites of caribou (genome accession GCA_019903745.1) and related ungulates. We obtained coding sequences (CDS) and peptide sequences of eight species: *Bos taurus*, *Oryx gazella*, *Capra aegagrus hircus*, *Equus caballus*, *Elaphurus davidianus*, *R. tarandus*, *Ovis aries*, and *Odocoileus virginianus* from the Ensembl Genome Browser and GIGA Science database (Table S1): these were selected because they had known divergence times (Chen et al., 2019). PorthoMCL (Tabari & Su, 2017) was used to identify orthologs in which we followed an eight‐step process to obtain the ortholog list shared between species. The orthologs were aligned using a customized *perl* script that implements the *pal2nal* aligner, following methods from Jeffares et al. (2015) with gaps removed.

The aligned ortholog fasta files were then concatenated using FASconCAT‐G (Kück & Meusemann, 2010) and converted to PHYLIP format. A maximum likelihood‐based phylogenetic tree was created with the program Randomized Axelerated Maximum Likelihood (RAxML/8; Stamatakis, 2014; Figure S1). RAxML was run using the PHYLIP file with the following parameters: ‐m GTRGAMMA ‐p 1349 ‐N 100 ‐o cow ‐n BEST ‐s. The tree was then calibrated in MCMCtree (Puttick, 2019) using known divergence times of 18.3–28.5 mya for cow‐sheep split and 3.9–8.1 mya for sheep‐goat split (Benton & Donoghue, 2007; Chen et al., 2019). The ratio of non‐synonymous to synonymous substitutions, d*N*/d*S*, was estimated for each branch using the *CODEML* function in *PAML/4.9* (Jeffares et al., 2015; Yang, 2007). The parameter *dS* approximates *μ* (Gillespie, 2004) since it reflects the number of substitutions that do not alter the protein sequence (Jeffares et al., 2015). To approximate a lineage specific *μ*, the *dS* value was divided by the estimated divergence time between species and by generation time of the focal species. For caribou, we used the generation time of 6 years (COSEWIC, 2017).

### 2.2 Individual sequence data

**FIGURE 1:**
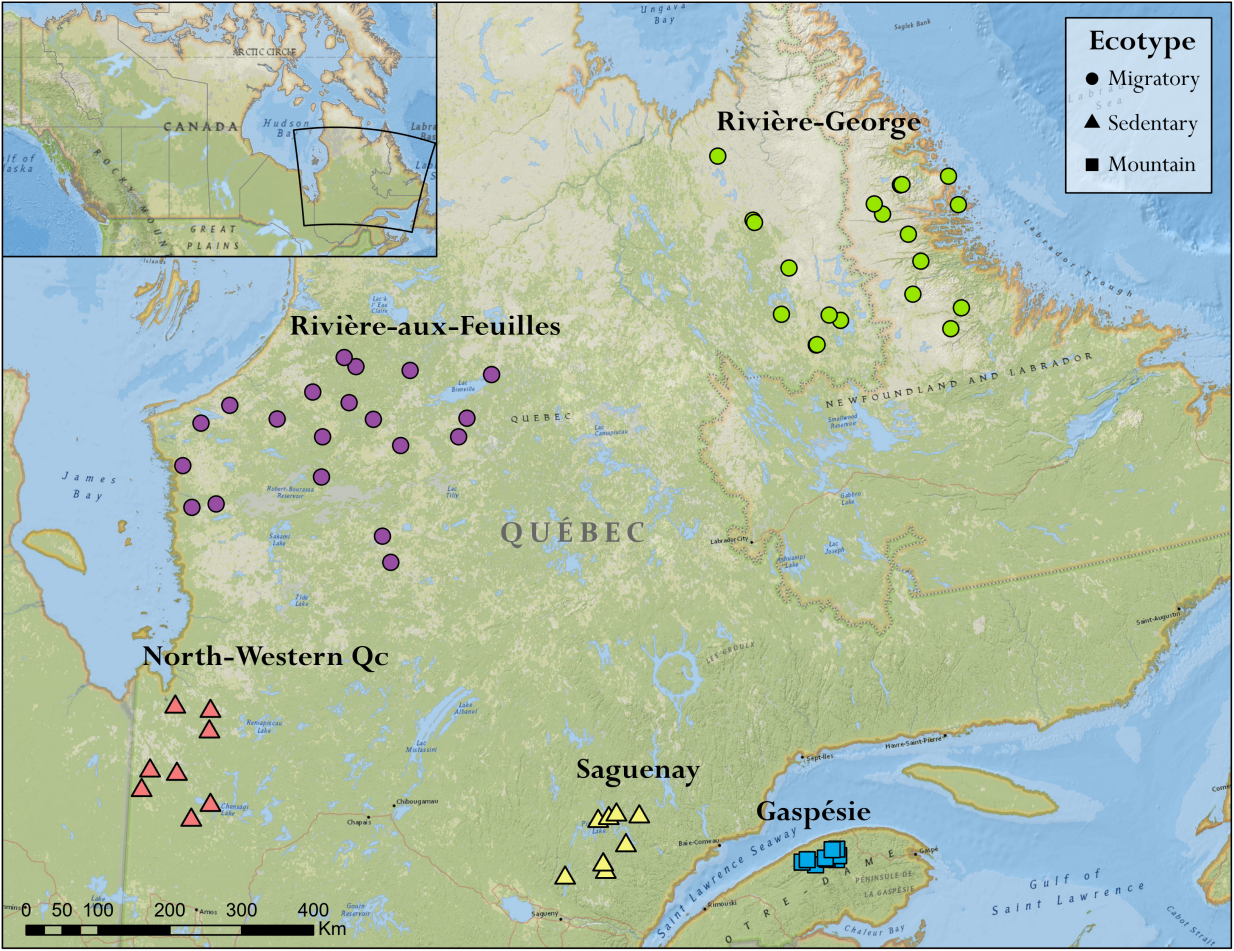
Geographical location of each individual caribou analyzed in this study (*n* = 8–20 per population, *n* total = 67). Five caribou populations and three ecotypes are represented

Caribou samples were collected from five populations (*n* = 67) across the province of Québec (Figure 1) and individuals were sequenced to ~5X whole genome coverage on an Illumina HiSeqX with paired‐end 150 bp reads. We aimed for a minimum sampling of eight individuals per population (Felsenstein, 2006; Nazareno et al., 2017). All data are deposited in the SRA (Accession SRP378572). These five populations consist of two migratory groups, Rivière‐aux‐Feuilles and Rivière‐George (TRAF and TRG respectively), two sedentary groups – North‐Western Qc and Southern Saguenay, and one mountain group – Gaspésie – (Figure 1). These populations were chosen because they are distributed throughout the caribou range in Québec and represent defined units with no major overlap. The mountain group is separated by the Saint Lawrence River, making it highly differentiated (Yannic et al., 2014) with a small (<100) comparative census size (*N*_C_). *N*_C_ values represented the estimated number of adults in the population and were collected from reported estimates via aerial surveys and population monitoring for Rivière‐aux‐Feuilles and Rivière‐George (Brodeur et al., 2018), North‐Western and Southern Saguenay (Szor & Brodeur, 2017). Estimates of contemporary *N*_e_ derived from microsatellites for our sampled herds range from 47 to 287, but values for the migratory herds could not be estimated (Yannic et al., 2016).

### 2.3 Population genomic data processing

Adaptors and other non‐target data were trimmed from the raw fastq files using Trimmomatic/0.36 (Bolger et al., 2014). Reads were mapped to the reference genome (GCA_019903745.1) using Samtools/1.12 and the Burrows‐Wheeler Aligner. Mapped reads were filtered for duplicates using Picard MarkDuplicates. Uniquely mapped reads were selected using sambamba/0.7.0, and we performed indel realignment using GATK/3.8 (Van der Auwera et al., 2013). Genotype likelihoods were estimated using ANGSD/0.933 (Korneliussen et al., 2014). We applied the following parameters: ‐doVcf 1 ‐doGeno 4 ‐doPost 1 ‐GL 2 ‐SNP_pval 1e‐6 ‐minMapQ 20 ‐minQ 20 ‐doCounts 1 ‐skipTriallelic 1 ‐doGlf 2, which uses the GATK model. We used Beagle 5.1 to convert GLs into hard‐calls (Browning et al., 2018) using impute = false overlap = 3000 window = 100,000 gprobs = TRUE parameters. The final VCF file was filtered for scaffolds larger than 100,000 bp.

### 2.4 Estimation of summary statistics

Genome wide *θ*_*π*_, Tajima's *D*, and *F*_ST_ were estimated using a sliding window approach in vcftools/0.1.13 (Danecek et al., 2011). Each of these estimates were estimated on genic regions, non‐genic regions, and the whole genome. Non‐genic regions are reported, but all estimates were highly correlated, with reduced diversity in genic regions (Table S2). *θ*_*π*_ and Tajima's *D* estimates were completed over a window size of 10,000 base pairs and run for each scaffold in the separate populations. Results for each scaffold were averaged over the entire population. *F* was estimated for each individual using the ‐‐het flag in PLINK/1.07. ROH were identified using a window‐based approach implemented in PLINK/1.07 from an input file generated by ANGSD (Korneliussen et al., 2014). Following methods used by Foote et al. (2021), we first generated a T‐ped file for each scaffold >10 Mb, and ran PLINK to identify the ROH for each scaffold with the following filters: ‐‐homozyg‐snp 15 ‐‐homozyg‐kb 100 ‐‐homozyg‐density 20 ‐‐homozyg‐gap 1000 ‐‐homozyg‐window‐snp 15 ‐‐homozyg‐window‐het 5 ‐‐homozyg‐window‐missing 5 ‐‐homozyg‐window‐threshold 0.05. Individual estimates of inbreeding were estimated by summing the ROH and dividing it by the total length of scaffolds in the analysis, to represent the proportion of the genome with ROH per individual (*F*_ROH_). *F*_ST_ was estimated for each population pair using ‐‐weir‐fst‐pop vcftools/0.1.13. Euclidean distance in kilometers was calculated between the centroid of each population range and isolation by distance was tested by running a Mantel test using the R package ecodist/2.0.7.

*θ*_*π*_ and *μ* estimates were input into the equation: *θ*_*π*_ *= 4 N*_e_ *μ* to solve for ancestral *N*_e_ of each population (also referred to as coalescent *N*_e_, see Peart et al., 2020). Contemporary estimates of *N*_e_ were calculated using the linkage‐disequilibrium (LD) equation of Waples (2006) based on genotype correlations (*R* ^2^) with the sample size correction of Waples et al. (2016). Because genome scale data can inflate *R* ^2^ (Waples et al., 2016), we generated 100 random VCF files each containing 10,000 random SNPs, estimated *R* ^2^ and applied ‐‐maf 0.05 filter using vcftools/0.1.13. We calculated the mean and standard deviation of contemporary *N*_e_ for each population.

### 2.5 Demographic inference

**FIGURE 2:**
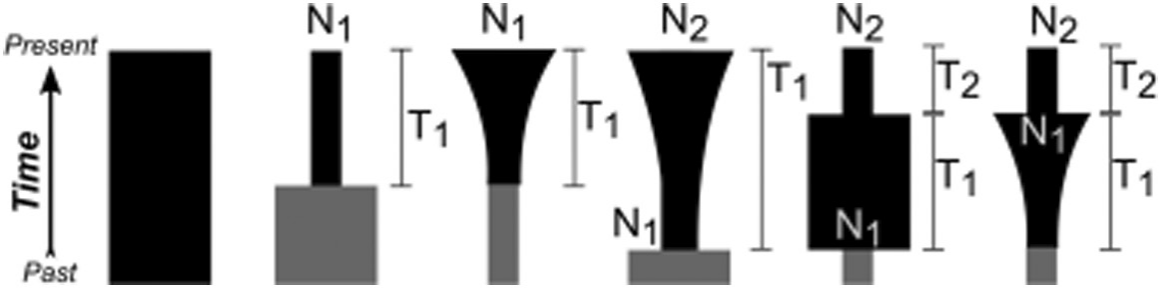
A visual of the demographic scenarios; (1) model 1 (N/a): Standard neutral model; (2) model 2 (*N*_1_, *T*): A single, instant change; (3) model 3 (*N*_1_, T): Gradual change; (4) model 4 (*N*_1_, *N*_2_, *T*_1_): Instantaneous size change followed by gradual change; (5) model 5 (*N*_1_, *N*_2_, *T*_1_, *T*_2_): Two independent, instant changes; and model 6 (*N*_1_, *N*_2_, *T*_1_, *T*_2_): Gradual change followed by an exponential distribution. The magnitude of change is parameterized as *N*_1_ and *N*_2_ (size relative to ancestral population size, depicted in grey). Modified from Peart et al. (2020).

Inference of demographic history for each population was based on the folded site frequency spectrum (SFS) and modelled using a diffusion‐based approach executed through Diffusion Approximation for Demographic inference (δaδi; Gutenkunst et al., 2009). Population projections for 1D SFS were generated from the VCF file using δaδi, to account for missing data and standardize the SFS (Marth et al., 2004). Six demographic scenarios were assessed for each population (Gutenkunst et al., 2010; Peart et al., 2020). The demographic models and the parameters are as follows (Figure 2):

1. **Constant population size**; Model 1 (N/A): Standard neutral model
2. **Single population change at time *T***_**1**_; Model 2 (*N*_1_, *T*): A single, instant change; Model 3 (*N*_1_, *T*): Gradual change.
3. **Two independent population size changes at *T***_**1**_ **and *T***_**2**_; Model 4 (*N*_1_, *N*_2_, *T*_1_): Instantaneous size change followed by gradual change; Model 5 (*N*_1_, *N*_2_, *T*_1_, *T*_2_) Two independent, instant changes; and Model 6 (*N*_1_, *N*_2_, *T*_1_, *T*_2_): Gradual change followed by an exponential change.

The optimum model with the lowest optimized log‐likelihood of all run models was identified, and 95% confidence intervals of each parameter based on 100 bootstrap replicates were calculated.

## 3 RESULTS

### 3.1 Mutation rate, μ

We extracted a total of 997 orthologs among eight species of related ungulates and generated a phylogenetic tree calibrated with known split times. It was estimated that caribou and *O. virginianus* split ~5.4 mya (Figure S1). The d*N*/d*S* ratio was <1 for each species in the tree, indicative purifying selection (Table S3). The mutation rate estimate with the generation time of 6 years was 3.46 × 10^−8^ mutations/site/generation (or 5.77 × 10^−9^ mutations/site/year), which is consistent with other mammal lineages (Kumar & Subramanian, 2002; Peart et al., 2020).

### 3.2 Detecting genetic variation and population structure

We analyzed 67 re‐sequenced caribou genomes that had an average final read depth of 4.2×. The total reads generated, and basic sample information can be observed in Table S4. After filtering for scaffold length, a total of 28,847,683 SNPs were identified across all individuals. A principal component analysis on all the individuals showed strong population structure according to ecotypes and provenance (Figure S2). Latitude appears to be the driving factor underlying this differentiation (*R* ^2^ = 0.46, *p* = 3.55 × 10^−10^, Figure S3). Diversity metrics confirmed that the Gaspésie mountainous group is highly differentiated from the other two ecotypes (*F*_ST_ > 0.1; Table S5). There was less genetic differentiation within ecotypes than between ecotypes (Table S5). No evidence for isolation by distance was detected (Mantel *R* = −0.1, *p* = 0.68, Figure S4), largely due to the St. Lawrence separating the Gaspésie from relatively close (geographically) populations.

### 3.3 Genome summary statistics

**FIGURE 3:**
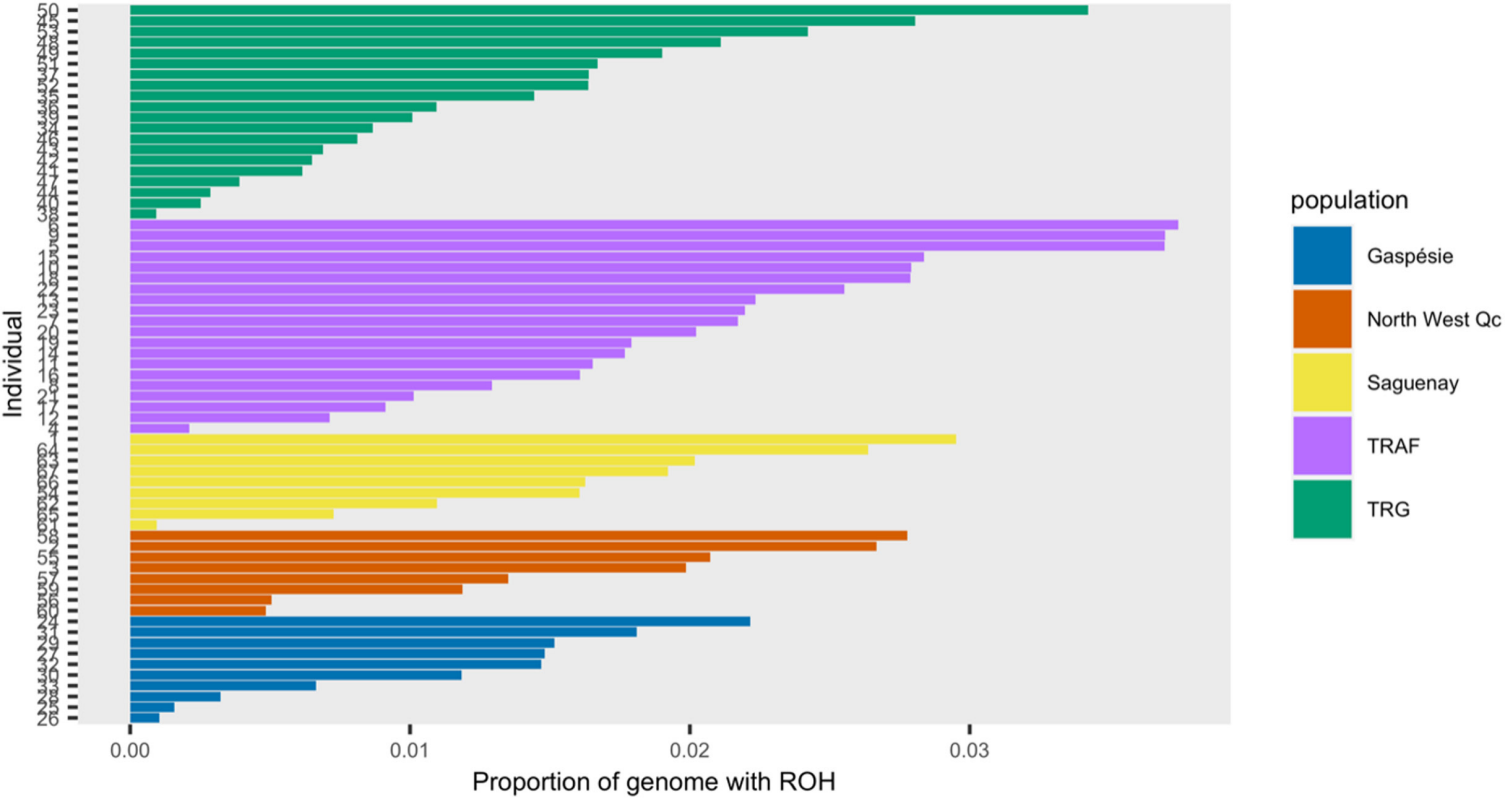
Proportion of the genome covered by runs of homozygosity (ROH) for each individual, separated by population. The average proportion by each individual is represented by *F*_ROH_ in Table 1. TRAF = Rivière‐aux‐Feuilles and TRG = Rivière‐George.

The calculated inbreeding coefficient, *F*, ranged from 0.09 to 0.31 with Gaspésie showing the highest *F* and the migratory populations showing the lowest (Table 1). The highest *F*_ROH_ were found in the migratory population, TRAF (Table 1). Gaspésie, notably, had the lowest *F*_ROH_, despite being the most isolated population (Figure 3). Genetic variation, shown as nucleotide diversity *θ*_*π*_, was higher in the non‐genic regions of the genome (see Supplemental for genic and whole‐genome diversity statistics). The Gaspésie population showed the least amount of nucleotide diversity among individuals (0.0019). The populations with the highest nucleotide diversity among individuals were the TRAF and TRG populations. Ancestral *N*_e_ ranged from 12,030 (Gaspésie) to 15,513 (TRAF; Table 2). Contemporary *N*_e_ ranged from 11 (Gaspésie) to 162 (TRG; Table 2). Each population displayed a small contemporary *N*_e_ to *N*_C_ ratio (Table 2). Summary statistics showed reduced coverage resulted in lower diversity estimates (Table S5).

**TABLE 1:** Inbreeding and ROH estimates of each pooled population

Caribou population	Mean *F*	Mean total length ROH (kb) per individual	Mean *F*_ROH_ per individual
TRAF	0.09	27,695.56	0.021
TRG	0.10	17,145.99	0.013
North‐Western Qc	0.13	21,646.99	0.016
Saguenay	0.16	21,669.41	0.016
Gaspésie	0.31	14,513.20	0.011

*Note*: *F* is shown as the average among the individuals sampled. ROH is shown as both the average length of total ROH among individuals in each population and the proportion of genome containing ROH (*F*_ROH_). TRAF = Rivière‐aux‐Feuilles and TRG = Rivière‐George.

**TABLE 2:** Population genetic diversity summary statistics, estimated adult census size, and *N*_e_/*N*_C_ ratio. TRAF = Rivière‐aux‐Feuilles and TRG = Rivière‐George

Caribou population	θ_π_ x 10^−3^	*N*_C_	Ancestral *N*_e_	Ancestral *N*_e_/*N*_C_	Contemporary *N*_e_	Contemporary *N*_e_/*N*_C_
TRAF	2.15	152,000	15,513	0.1	123	0.001
TRG	2.13	4200	15,399	3.7	162	0.04
North‐Western Qc	2.12	259	15,345	59.3	42	0.16
Saguenay	2.03	175	14,690	83.9	18	0.1
Gaspésie	1.67	80	12,030	150.4	11	0.12

### 3.4 Tajima's *D* and demographic changes

**FIGURE 4:**
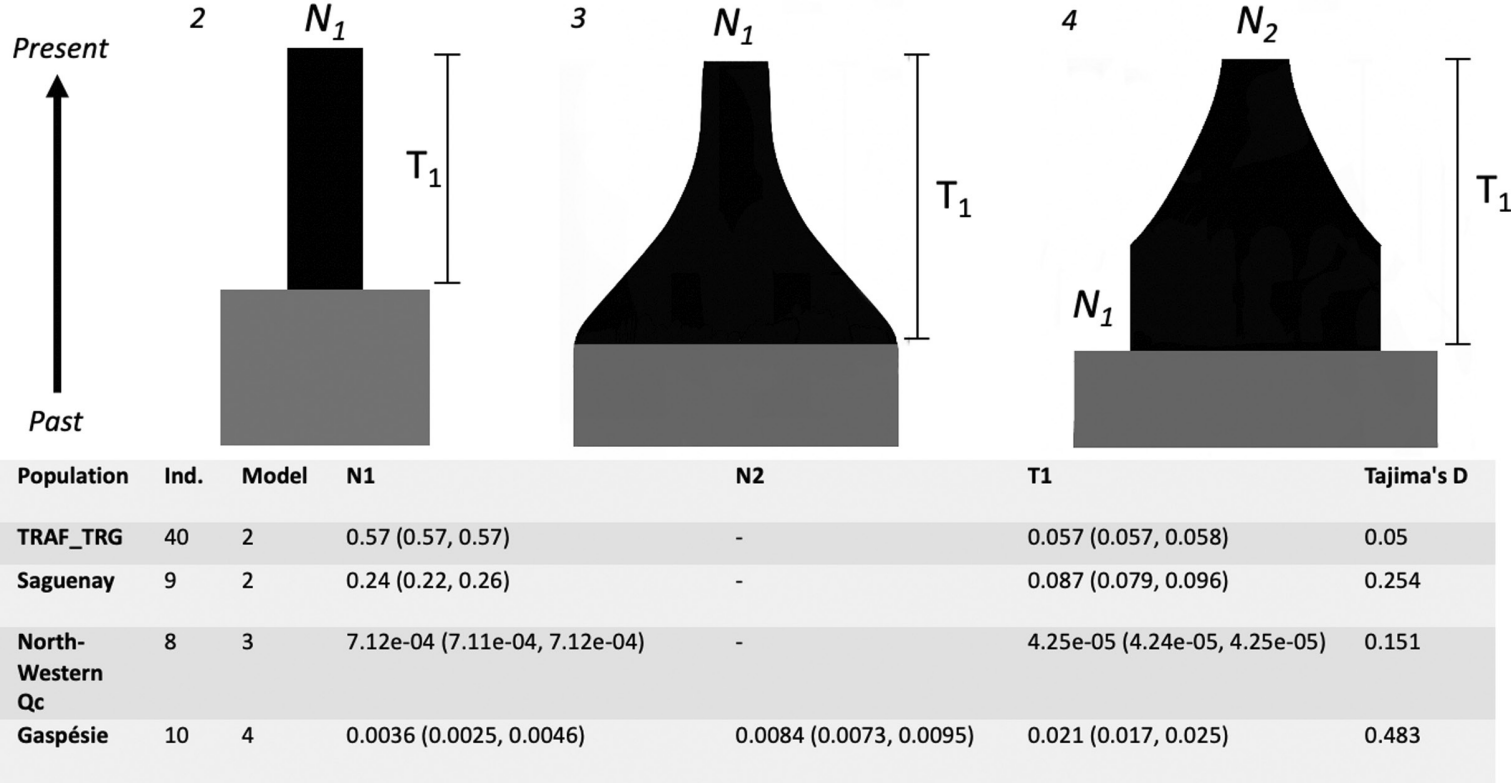
Summary of best fitting single population (1D) model and demographic parameter estimates from δaδi for each caribou population with 95% CI. Parameter estimates are *N*_1_ and *N*_2_ as the magnitude of population change, and timepoints of change as T1 (in 2*N*_e_ generations). Tajima's *D* indicates population decline for all populations. Ind represents the number of samples used for the analyses. TRAF = Rivière‐aux‐Feuilles and TRG = Rivière‐George.

Based on the PCA (Figure S3), TRAF and TRG were merged for the demographic inference (note the same was done in Yannic et al., 2016). We explored the relationship between contemporary and ancestral *N*_e_/*N*_C_ and demographic change. Tajima's *D* estimates were all >0, indicating a deficit of rare alleles, consistent with a historical bottleneck (Figures 4 and S6). Demographic parameter estimates, as inferred from δaδi, suggest the two migratory groups and one sedentary group experienced model 2 (instant population change or a bottleneck) was the best fitting model (Figure 4). A more recent, gradual population change was seen in the North‐West population based off *T* estimates in Figure 4. The Gaspésie population is predicted to have experienced two shifts in population size; an instantaneous reduction with subsequent gradual reduction of population size, indicative of an overall 98% reduction in *N*_e_.

## 4 DISCUSSION

Several caribou populations in Québec, Canada, have experienced recent declines, with some populations now being federally listed as endangered by provincial and federal governments (e.g. COSEWIC, 2017). Province‐wide sampling of genomes provided a holistic approach to investigating the genomic signatures of populations within Québec. Our comparative population genomics assessment evaluated the interplay between demographic events and genomic diversity, and we found that each population is experiencing a loss of rare alleles and has undergone varying degrees of demographic decline (i.e. Tajima's *D* > 0 and *N*_1_ < 1, Figure 4). However, there was no strong signature of inbreeding associated with diversity or demographic estimates, and only the Gaspésie decline was shown to be recent in timing (i.e. *T* equal to *2N*_e_ generations in Figure 4). High ancestral *N*_e_/*N*_C_ signal, and not contemporary *N*_e_, identified the populations at highest risk of extirpation and extinctions (Peart et al., 2020); this pattern would apply to our study as the Gaspésie and Saguenay populations are considered particularly vulnerable.

### 4.1 Population structure and inbreeding

The minimal amount of differentiation between the two migratory caribou populations, Rivière‐aux‐Feuilles and Rivière‐George (TRAF and TRG respectively; *F*_ST_ = 0.0027), is concordant with previous genetic studies, showing high gene flow between these two populations (Boulet et al., 2007; Yannic et al., 2016). Geographical distance separating the sedentary populations limits the amount of gene flow, however it does not limit the migratory populations from overlapping and interacting with the northernmost sedentary populations (Boulet et al., 2007; Yannic et al., 2016). The largest *F*_ST_ values were observed between the Gaspésie population and all other populations (*F*_ST_ = 0.14–0.15), which is expected as it has the smallest range, and the St. Lawrence River has generated prolonged isolation. The relationship of genomic variance to latitude (Figure S3), would be consistent with a northern recolonization following the retreat of the icesheets starting some twenty‐thousand years ago (Yannic et al., 2014).

We observed similar amounts of inbreeding in the two migratory populations (*F* = 0.09 and 0.1), and the highest observed inbreeding rate was observed in the Gaspésie population (*F* = 0.31). An increased level of inbreeding can be a product of population declines and isolation, which is consistent with southern populations (Tables 1 and 2). Interestingly, *F*_ROH_ varies only slightly between populations; increased ROH are expected in smaller, isolated populations, that have undergone the most recent bottlenecks (e.g. Palkopoulou et al., 2015; Solmundson et al., 2020). It is possible that the ROH detected in the larger populations might actually be under selection (e.g. Ilmonen et al., 2007; Marras et al., 2015); although this would require further assessment. Most likely is simply ROH have not had enough time to accrue given the recent collapse in *N*_C_ across the province is not detectable in the demographic models (Figure 2). While inbreeding is of future concern in low *N*_C_ populations, current genetic diversity measures do not suggest it is an immediate problem. In fact, many populations with low *N*_C_ and inbreeding have nuanced patterns of inbreeding depression (Pekkala et al., 2014), with unclear links to population viability (Teixeira & Huber, 2021). The relationship between deleterious genetic signatures and *N*_C_ seems dependent, in part, on time (van der Valk et al., 2021), meaning it is not necessarily predicted that caribou in Quebec would have high levels of ROH or deleterious alleles at this stage given genome‐wide demographic signatures predate crashes in *N*_C_.

### 4.2 Contemporary and ancestral *N*_e_ and the relationship to *N*_C_

The ancestral and contemporary *N*_e_ values reflect different time scales, and accordingly the impact of drift and selection. Ancestral estimates are incorporating genetic information scaling back to the most recent common ancestor and capture evolutionary forces shaping genetic variation over the order of *4N*_e_ generations. Despite the clear population structure (Figure S2), the similar ancestral *N*_e_ values among populations suggest a relatively large amount of shared standing genetic variation across Québec populations, consistent with PSMC analyses that show near identical trajectories across many populations (Taylor et al., 2021). Similar trends have been seen in other wild populations such as the vinous‐throated parrotbill (*Sinosuthora webbiana*; Lai et al., 2019) and crows (*Corvus*), flycatchers (*Tyrannidae*), and finches (*Fringillidae*; Vijay et al., 2017). The contemporary *N*_e_ values while much smaller, follow similar patterns as the ancestral values – TRAF and TRG having the largest *N*_e_ and Gaspésie having the smallest *N*_e_ – with a slightly wider range when put to scale. This same genetic risk is seen in other mammal populations which experienced a population bottleneck (e.g. Houlden et al., 1996; Tokarska et al., 2009); however, none of these low *N*_e_ and *N*_C_ caribou populations have high degrees of inbreeding compared to other endangered species (von Seth et al., 2021), suggesting they also have yet to be impacted by isolation and numerical decline. Importantly, it is the ancestral *N*_e_:*N*_C_ ratio, not contemporary, that flags the southern populations as conservation concern, which is consistent with the stochastic and ambiguous contemporary *N*_e_:*N*_C_ ratios as it pertains to conservation (Palstra & Ruzzante, 2008). Another important observation is our contemporary *N*_e_ values are considerably lower (<<50%) than estimates of Yannic et al. (2016) for the same herds. LD‐based estimates are typically based on genotype correlations (*r* ^2^), with the discrepancy here likely due to reduced *r* ^2^ values in microsatellite data (and hence inflated *N*_e_ value), as these markers are typically selected based on linkage disequilibrium and hypervariability.

The actual size of the population, as well as contemporary *N*_e_ estimates can change drastically in the course of a few generations (England et al., 2010; Ferchaud et al., 2016) and thus we can expect current anthropogenic activities to continue to have a strong impact on *N*_C_ (Plante et al., 2020) and contemporary *N*_e_ (Stoffel et al., 2018), but less so on ancestral *N*_e_ (Peart et al., 2020). As concluded by Peart et al. (2020), a high ancestral *N*_e_/*N*_C_ ratio (>1) is consistent with recent bottlenecks, significantly reducing current‐day *N*_C_, while having little effect on the ancestral, long‐term *N*_e_ or nucleotide diversity. The ancestral *N*_e_/*N*_C_ ratio and demographic models (all showing a historic declines) are strongly correlated to Tajima's *D* (this study; Peart et al., 2020), suggesting that Tajima's *D* an easily generated metric that is informative for conservation.

### 4.3 The conservation genomics toolbox

Ultimately, the population statistics measured in this study are all metrics of diversity reflecting past demographic events that are used to gauge the future adaptive potential. The analytical toolbox has expanded to incorporate genotype likelihoods (e.g. Çelik & Tuncali, 2022), allowing for more samples at lower coverage to be integrated into sampling design, recognizing some metrics based‐off hard calls will be impacted (Benjelloun et al., 2019). Thus, we suggest a holistic conservation genomics view should assess demographic history to inform management and conservation decisions as, for example, *F*_ROH_ were not necessarily informative for assessing a population's current or ancestral genetic diversity (see also von Seth et al., 2021). Contemporary *N*_e_/*N*_C_ might be also misleading (Ferchaud et al., 2016; Palstra & Ruzzante, 2008) as it does not consider the long‐term population dynamics and ancestral genomic variation each population harbours.

The majority of governing bodies use *N*_C_ values as their main source of population data (IUCN Standards and Petitions Committee, 2019), which can be difficult to obtain and disruptive to the population. Here we argue for the importance of ancestral *N*_e_ and Tajima's *D* given they are derivated from genome‐wide metrics of diversity and there is clear connection to demographic history and conservation status. This approach has fundamental implications for understanding populations which have not been intensely studied and recently discovered species that do not have accurate census data or population histories. Diversity estimates alone correlate to life history tactics (Ellegren & Galtier, 2016), and thus we can make some inference as to conservation status with Tajima's *D* (this study; Peart et al., 2020). While Québec caribou populations are either declining or of concern, they might still harbour enough ancestral genetic variation to replenish, if conservation decisions are made in favour of these populations, specifically supporting *N*_C_. The Gaspésie and Saguenay populations should be a management priority. The genetic signatures are still relatively positive and suggest that if the time spent in the current bottlenecks is minimized, so too will be the impact on genomic diversity and adaptive potential.

## CONFLICT OF INTEREST

The authors declare no conflicts of interest.

## BENEFITS GENERATED

Benefits GeneratedBenefits from this research accrue from the sharing of our data and results on public databases as described above.

## Supporting information


Appendix S1

## Data Availability

All bioinformatic and analytical code available on GitLab (https://gitlab.com/WiDGeT_TrentU). All sequence data have been uploaded to the SRA (SRP378572).
