# Supplementary material for: Demographic history and conservation genomics of caribou (Rangifer tarandus) in Québec
Source: Evol Appl. 2022 Oct 25;15(12):2043–53. doi: 10.1111/eva.13495 (PMC9753816; doi:10.1111/eva.13495)
Supplement: Supplementary file 1 — Appendix S1 [file EVA-15-2043-s001.docx]

# **Supplementary**

Table S1. Genome accession numbers and references for ungulates used in the ortholog alignment (n=8).

| Species | Genome accession number/reference |
| --- | --- |
| Capra aegagrus hircus | GCA_001704415.1 |
| Ovis aries | GCA_002742125.1 |
| Oryx gazella | Farré et al., 2019 |
| Bos taurus | GCA_002263795.2 |
| Odocoileus virginianus | Sabury et al., 2011 |
| Rangifer tarandus | GCA_019903745.1 |
| Elaphurus davidianus | Zhang et al., 2018 |
| Equus caballus | GCA_002863925.1 |

Table S2. Nucleotide diversity (θ_π_) and Tajima's D for the whole genome and the genic regions of each caribou population. TRAF = Rivière-aux-Feuilles and TRG = Rivière-George.

|  | Whole genome | | Genic | | |
| --- | --- | --- | --- | --- | --- |
| **Population** | θ_π_ x 10^-3^ | Tajima's *D* |  | θ_π_ x 10^-3^ | Tajima's *D* |
| **Saguenay** | 2.17 | 0.255 |  | 0.603 | 0.078 |
| **North-Western Qc** | 2.265 | 0.152 |  | 0.624 | 0.047 |
| **TRAF** | 2.291 | 0.106 |  | 0.611 | 0.041 |
| **Gaspésie** | 1.78 | 0.486 |  | 0.519 | 0.139 |
| **TRG** | 2.27 | 0.094 |  | 0.607 | 0.036 |

Table S3. Summary of values produced from Phylogenetic Analysis by Maximum Likelihood (PAML) to infer mutation rate. dN = non-synonymous, dS = synonymous, t = time.

| Species | dN/dS | dN | dS | t |
| --- | --- | --- | --- | --- |
| Capra aegagrus hircus | 0.2102 | 0.0036 | 0.0172 | 0.020 |
| Ovis aries | 0.3824 | 0.0114 | 0.0299 | 0.047 |
| Oryx gazella | 0.3858 | 0.0189 | 0.0491 | 0.078 |
| Bos taurus | 0.1601 | 0.0078 | 0.0486 | 0.052 |
| Odocoileus virginianus | 0.2858 | 0.0071 | 0.025 | 0.034 |
| Rangifer tarandus | 0.4773 | 0.0149 | 0.0312 | 0.056 |
| Elaphurus davidianus | 0.4779 | 0.0326 | 0.0682 | 0.123 |
| Equus caballus | 0.1616 | 0.0482 | 0.2979 | 0.319 |

Table S4. A summary of metadata from the sampled individuals. The individual caribou ID, ecotype, population assignation, sampling date, sex, age class and number of reads of the genome are displayed. TRAF = Rivière-aux-Feuilles and TRG = Rivière-George.

| ID-GAPP | Ecotype | Region | Sampling Date | Sex | Age Class | Number of Reads |
| --- | --- | --- | --- | --- | --- | --- |
| GAP-073 | Sedentary | Saguenay | 2018-02-17 | F | Adult | 87042130 |
| GAP-103 | Sedentary | N-du-Qc-West | 2018-03-29 | F | Adult | 87243856 |
| GAP-104 | Sedentary | N-du-Qc-West | 2018-03-29 | M | Adult | 68396926 |
| GAP-105 | Migratory | TRAF | 2016-03-22 | M | Adult | 63959538 |
| GAP-106 | Migratory | TRAF | 2016-03-24 | F | Adult | 87274138 |
| GAP-107 | Migratory | TRAF | 2016-03-26 | M | Adult | 84947714 |
| GAP-108 | Migratory | TRAF | 2016-03-27 | F | Adult | 69805054 |
| GAP-109 | Migratory | TRAF | 2016-03-28 | M | Adult | 65378878 |
| GAP-110 | Migratory | TRAF | 2016-04-26 | F | Adult | 93609684 |
| GAP-111 | Migratory | TRAF | 2017-03-03 | F | Adult | 83212546 |
| GAP-112 | Migratory | TRAF | 2017-03-05 | M | Adult | 77700368 |
| GAP-113 | Migratory | TRAF | 2017-03-06 | M | Adult | 61502344 |
| GAP-114 | Migratory | TRAF | 2017-03-08 | M | Adult | 82235404 |
| GAP-115 | Migratory | TRAF | 2017-03-09 | M | Adult | 80242588 |
| GAP-116 | Migratory | TRAF | 2017-03-23 | F | Adult | 89903826 |
| GAP-117 | Migratory | TRAF | 2017-03-29 | F | Adult | 77745714 |
| GAP-118 | Migratory | TRAF | 2017-03-30 | F | Adult | 73157878 |
| GAP-119 | Migratory | TRAF | 2018-02-15 | F | Adult | 82005028 |
| GAP-120 | Migratory | TRAF | 2018-02-15 | M | Adult | 83553488 |
| GAP-122 | Migratory | TRAF | 2018-02-22 | M | Adult | 85160152 |
| GAP-123 | Migratory | TRAF | 2018-02-22 | M | Adult | 70485546 |
| GAP-124 | Migratory | TRAF | 2018-03-13 | F | Adult | 84770156 |
| GAP-125 | Migratory | TRAF | 2018-03-14 | M | Adult | 84618928 |
| GAP-128 | Mountain | Gaspésie | 2013-02-08 | F | Adult | 42552518 |
| GAP-129 | Mountain | Gaspésie | 2013-02-10 | F | Adult | 31351685 |
| GAP-130 | Mountain | Gaspésie | 2013-02-10 | M | Adult | 27350658 |
| GAP-131 | Mountain | Gaspésie | 2013-02-11 | M | Adult | 41163364 |
| GAP-132 | Mountain | Gaspésie | 2013-02-15 | M | Adult | 31258420 |
| GAP-133 | Mountain | Gaspésie | 2013-02-15 | F | Adult | 43733224 |
| GAP-135 | Mountain | Gaspésie | 2014-02-21 | F | Adult | 41198070 |
| GAP-136 | Mountain | Gaspésie | 2014-02-23 | M | Adult | 44680493 |
| GAP-137 | Mountain | Gaspésie | 2014-02-26 | F | Adult | 43391254 |
| GAP-138 | Mountain | Gaspésie | 2014-02-26 | M | Adult | 35714436 |
| GAP-139 | Migratory | TRG | 2016-02-19 | F | Adult | 72016106 |
| GAP-140 | Migratory | TRG | 2016-02-20 | F | Adult | 78766252 |
| GAP-141 | Migratory | TRG | 2016-02-23 | M | Adult | 77215468 |
| GAP-142 | Migratory | TRG | 2016-02-25 | F | Adult | 82286514 |
| GAP-143 | Migratory | TRG | 2016-02-25 | F | Adult | 51316246 |
| GAP-144 | Migratory | TRG | 2016-02-25 | F | Adult | 74213378 |
| GAP-145 | Migratory | TRG | 2017-04-13 | F | Adult | 55943114 |
| GAP-146 | Migratory | TRG | 2017-04-13 | M | Adult | 56998212 |
| GAP-147 | Migratory | TRG | 2017-04-13 | F | Adult | 59807738 |
| GAP-148 | Migratory | TRG | 2017-04-13 | M | Adult | 56749426 |
| GAP-150 | Migratory | TRG | 2017-04-16 | M | Adult | 49325620 |
| GAP-151 | Migratory | TRG | 2017-12-11 | M | Adult | 87851628 |
| GAP-153 | Migratory | TRG | 2017-12-13 | M | Adult | 73726918 |
| GAP-154 | Migratory | TRG | 2017-12-13 | F | Adult | 59286288 |
| GAP-155 | Migratory | TRG | 2017-12-13 | M | Adult | 81313924 |
| GAP-156 | Migratory | TRG | 2017-12-18 | F | Adult | 84427762 |
| GAP-157 | Migratory | TRG | 2017-12-18 | M | Adult | 94039530 |
| GAP-158 | Migratory | TRG | 2017-12-19 | F | Adult | 78278206 |
| GAP-159 | Migratory | TRG | 2017-12-19 | M | Adult | 80818072 |
| GAP-161 | Migratory | TRG | 2017-12-19 | F | Adult | 87098254 |
| GAP-162 | Sedentary | Saguenay | 2018-02-14 | F | Adult | 82251168 |
| GAP-169 | Sedentary | N-du-Qc-West | 2018-03-26 | M | Adult | 84786218 |
| GAP-170 | Sedentary | N-du-Qc-West | 2018-03-26 | M | Adult | 64786908 |
| GAP-171 | Sedentary | N-du-Qc-West | 2018-03-28 | M | Adult | 80158986 |
| GAP-172 | Sedentary | N-du-Qc-West | 2018-03-28 | M | Adult | 91668654 |
| GAP-175 | Sedentary | N-du-Qc-West | 2016-03-18 | F | Adult | 65993900 |
| GAP-176 | Sedentary | N-du-Qc-West | 2016-03-20 | F | Adult | 58436152 |
| GAP-177 | Sedentary | Saguenay | 2018-02-13 | M | Adult | 45331166 |
| GAP-184 | Sedentary | Saguenay | 2018-02-17 | F | Adult | 75430528 |
| GAP-186 | Sedentary | Saguenay | 2018-02-17 | M | Adult | 83115914 |
| GAP-187 | Sedentary | Saguenay | 2018-02-19 | M | Adult | 84755692 |
| GAP-188 | Sedentary | Saguenay | 2018-02-19 | F | Adult | 61741464 |
| GAP-191 | Sedentary | Saguenay-Centre | 2018-02-13 | M | Adult | 80283078 |
| GAP-192 | Sedentary | Saguenay-Centre | 2018-02-14 | F | Adult | 77751198 |

Table S5. Matrix showing the F_ST_ between caribou populations (top half) and the centroid distance in kilometers of their distribution (bottom half). TRAF = Rivière-aux-Feuilles and TRG = Rivière-George.

|  | TRAF | TRG | Saguenay | North-Western Qc | Gaspésie |
| --- | --- | --- | --- | --- | --- |
| TRAF |  | 0.0027 | 0.044 | 0.031 | 0.14 |
| TRG | 632.7 km |  | 0.043 | 0.029 | 0.14 |
| Saguenay | 1009 km | 920.8 km |  | 0.026 | 0.15 |
| North-Western Qc | 854.7 km | 1152 km | 607.2 km |  | 0.14 |
| Gaspésie | 1137 km | 870.5 km | 290 km | 892 km |  |

Table S6. Population genetic diversity summary statistics from only the Gaspésie population samples including all data (100%) aand down-sampled to 75%, 50%, and 25% (~3X, ~2X and ~1X coverage respectively). Note SNPs were called using the ANGSD pipeline on just Gaspésie samples for this analysis. Full denotes the metrics from the manuscript where SNPs were called on all samples (i.e. all herds), but summary statistics estimated for each population separately.

| % of data used | Mean total length  ROH (kb) per individual | Mean F_ROH_ per individual | θ_π_ x 10^-3^ | Tajima’s D |
| --- | --- | --- | --- | --- |
| Full | 14513.20 | 0.011 | 1.67 | 0.48 |
| 100% | 13867.57 | 0.010 | 1.46 | 0.20 |
| 75% | 4339.72 | 0.0033 | 1.34 | 0.19 |
| 50% | 738.14 | 0.00066 | 1.11 | 0.28 |
| 25% | 212.61 | 0.00016 | 0.65 | 0.65 |


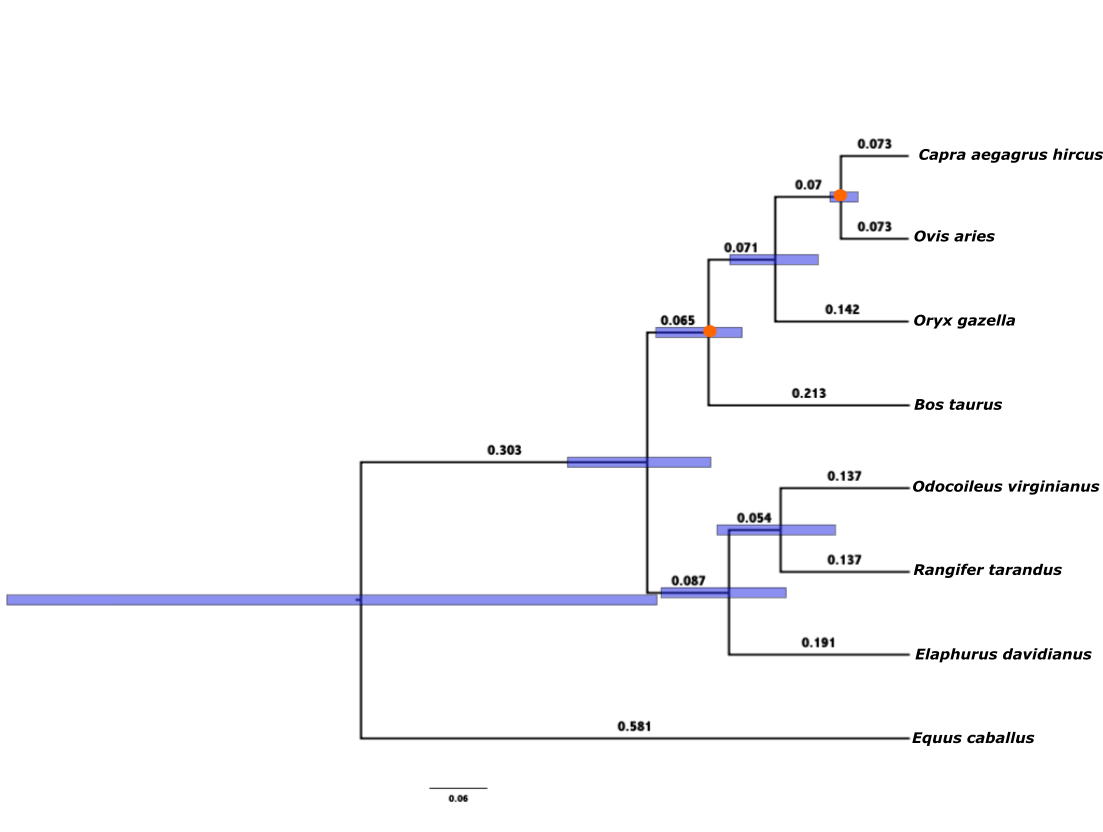


Figure S1. Maximum likelihood phylogenetic tree and divergence times. Purple lines represent 95% confidence intervals. Tree was calibrated using known split times of Bos taurus from Ovis aries and Ovis aries from Capra aegagrus hircus, as indicated by the orange dots.


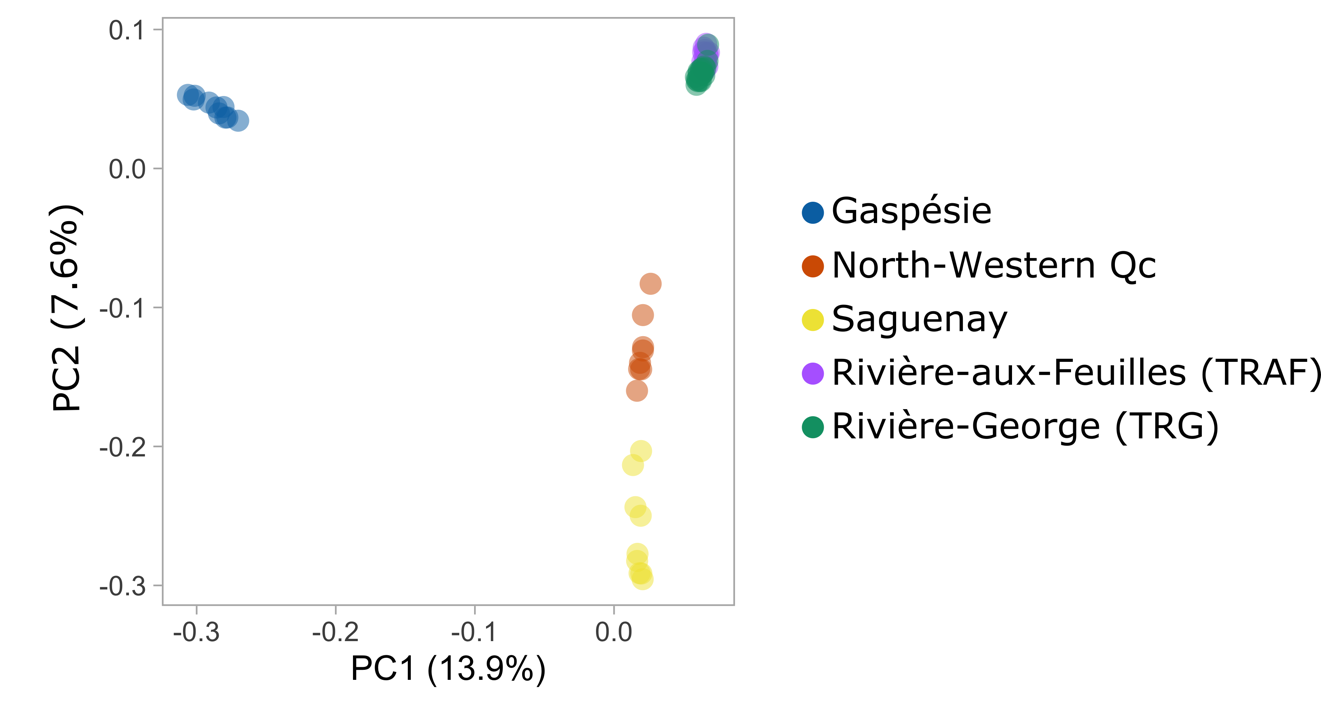


Figure S2. A plot of PC1 and PC2 from a Principal Component Analysis (PCA) on the five caribou populations in this study. Strong population structure within ecotypes is shown, with PC1 and PC2 explaining 21.5% of the genomic variation.


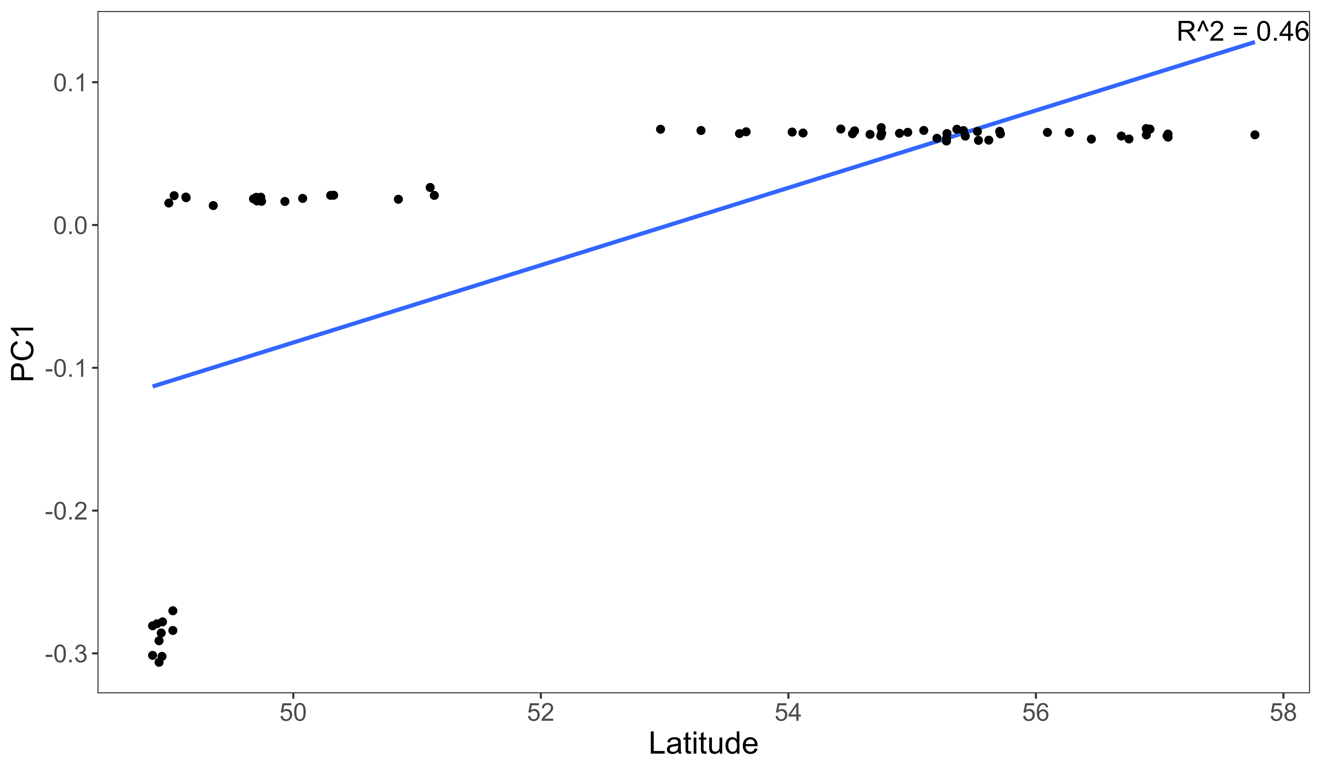


Figure S3*.* Linear regression suggests the relationship between PC1 from the principal component analysis and latitude was the highest explanatory variable (over latitude and PC2) of the differentiation between populations of caribou.


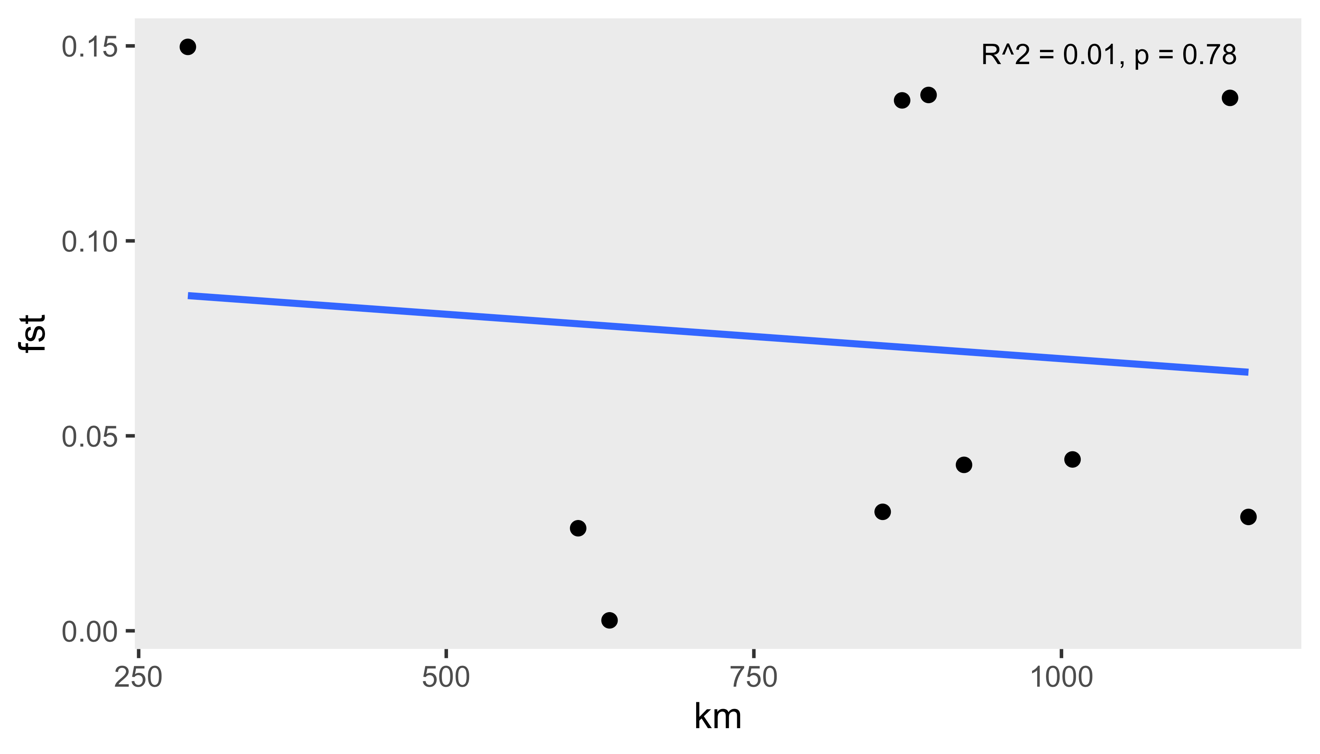


Figure S4. Plot of Mantel test for isolation by distance of caribou populations, using Euclidean distance from the geographical centroid of the study samples. Regression line shows no correlation (R^2^ = 0.01, p = 0.78).


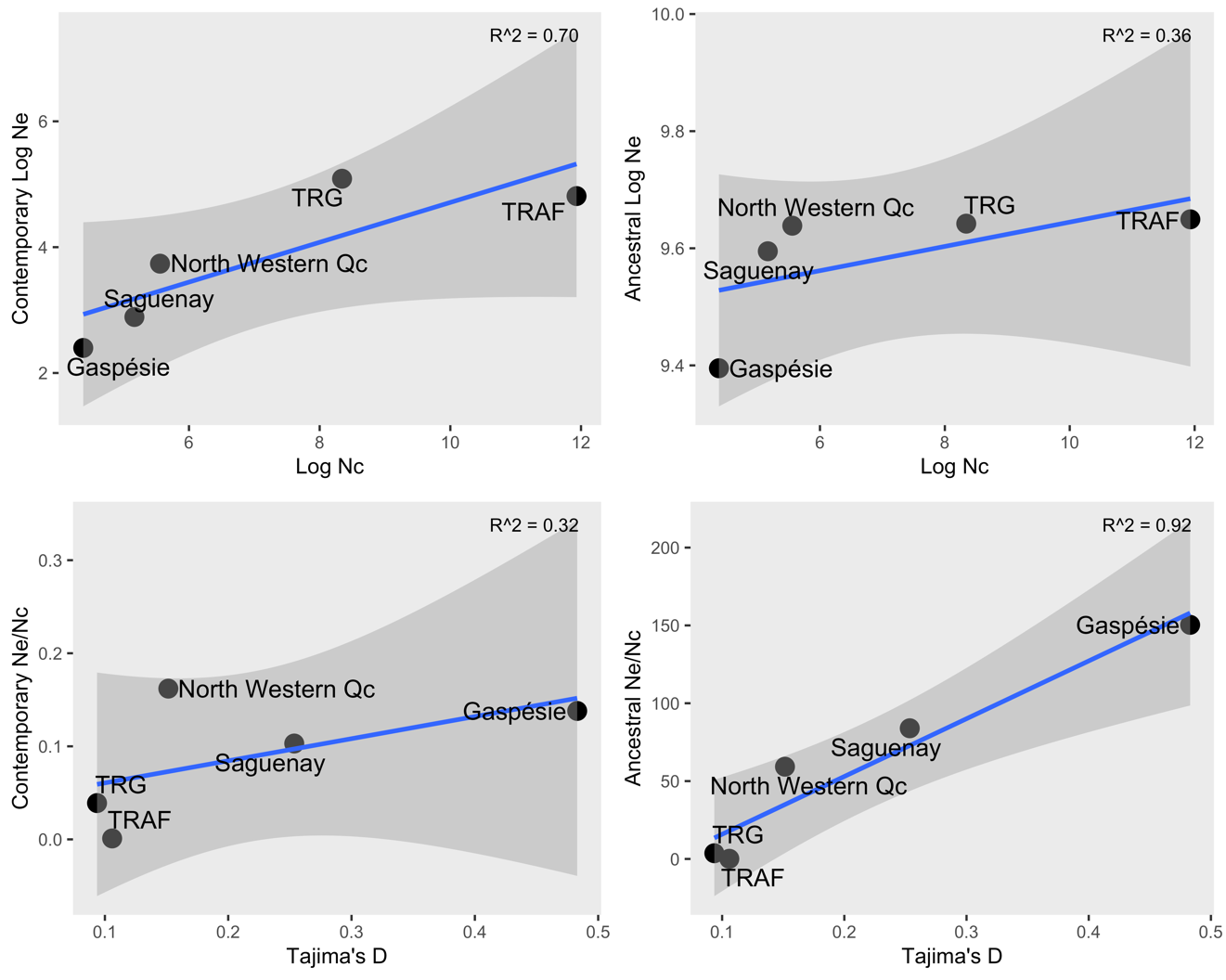


Figure S5. Relationships between A) contemporary N_e_ and B) ancestral N_e_ with N_C_ on a logarithmic base 10 scale. The blue line depicts regression, and the grey shaded area represents 95% CI. TRAF = Rivière-aux-Feuilles and TRG = Rivière-George. Relationships between C) contemporary Ne/NC and D) ancestral N_e_ / N_C_ and Tajima's D (reflecting the demographic history) for each population.
